# Supplementary material for: An Early Mediterranean-Based Nutritional Intervention during Pregnancy Reduces Metabolic Syndrome and Glucose Dysregulation Rates at 3 Years Postpartum
Source: Nutrients. 2023 Jul 22;15(14):3252. doi: 10.3390/nu15143252 (PMC10383706; doi:10.3390/nu15143252)
Supplement: Supplementary file 1 [file nutrients-15-03252-s001.zip › nutrients-2521647-supplementary.pdf]

Supplementary Table S1. Clinical and metabolic characteristics during pregnancy of analyzed women.

	CG N=369	IG N= 1031	P
**WEIGHT GAIN AT 12 GW (Kg)**	1.9 (0.1–3.3)	1.7 (0.3–3.4)	0.572
**WEIGHT GAIN AT 24-28 GW (Kg)**	7.3 (5.3–9.7)	6.9 (4.7–9.1)	0.040
**WEIGHT GAIN AT 36-38 GW (Kg)**	11.2 (8.1–14.1)	11.8 (8.8–15.0)	0.141
**SYSTOLIC BP / DIASTOLIC BP (mmHg)**			
**AT 12 GW**	109 (101–115)	108 (101–116)	0.889
	66 (60–73)	67 (61–73)	0.334
**AT 24 GW**	104 (97–112)	106 (98–113)	0.066
	62 (57–68)	63 (58–69)	0.108
**AT 36 GW**	111 (103–119)	113 (105–122)	0.005
	70 (65–76)	71 (65–78)	0.023
**FASTING BLOOD GLUCOSE (mg/dL)**			
**AT 12 GW**	81 (77–86)	81 (76–85)	0.075
**AT 24 GW**	86 (82–90)	84 (80–89)	0.002
**AT 36 GW**	77 (73–82)	76 (72–81)	0.050
**HOMA-IR**			
**AT 12 GW**	0.6 (0.3–1.3)	0.6 (0.3–1.6)	0.195
**AT 24 GW**	1.7 (1.1–2.6)	1.6 (1.1–2.3)	0.06
**AT 36 GW**	1.6 (1.1–2.6)	1.6 (1.0–2.5)	0.235
**HbA1C (%) AT 12 GW**	5.2 (5.0–5.3)	5.2 (5.0–5.3)	0.905
**24 GW**	5.0 (4.8–5.2)	4.9 (4.7–5.1)	0.000
**36 GW**	5.3 (5.1–5.5)	5.2 (5.0–5.4)	0.000
**CHOLESTEROL (mg/dL) AT 12 GW**	174 (155–196)	171 (151–192)	0.068
**24 GW**	246 (217–275)	243 (219–270)	0.594
**36 GW**	277 (236–314)	265 (236–296)	0.014
**TRIGLYCERIDES (mg/dL) AT 12 GW**	73 (59–99)	71 (56–96)	0.159
**24 GW**	149 (121–185)	152 (119–190)	0.601
**36 GW**	228 (190–277)	218 (174–270)	0.052
**GDM AT 24-28 GW N (%)**	88 (23.9%)	202 (19.5%)	0.05
**MEDICAL NUTRITION THERAPY**	53 (61.6%)	111 (57.8%)	0.202
**INSULIN TREATMENT**	45 (38.4%)	91 (42.2)	
**ECLAMPSIA**	2 (0.7%)	15 (1.3%)	0.300
**GESTATIONAL HYPERTENSION**	7 (2.3%)	37 (4.2%)	0.169
**NB >P90/<P10**	13 (3.5%)/20 (5.4%)	37(3.6%)/60(5.8%)	0.551 /0.446
**12 GW TSH µUI/ML**	1.9 (1.1–2.8)	1.9 (1.2–2.8)	0.642
**FT4 PG/ML**	8.5(7.7–9.4)	8.6 (7.8–9.5)	0.103
**24 GW TSH µUI/ML**	1.7 (1.2–2.5)	1.9 (1.3–2.5)	0.266
**FT4 PG/ML**	6.8 (6.2–7.7)	6.9 (6.2–7.6)	0.578
**36 GW TSH µUI/mL**	1.4 (0.9–2.1)	1.5 (1.0–2.1)	0.225
**FT4 pg/mL**	7.0 (6.3–7.8)	6.9 (6.2–7.8)	0.686
**MEDAS SCORE**			
**12 GW**	5 (3;6)	5 (4; 6)	0.09
**24 GW**	5 (4;6)	6 (5; 7)	0.0000
**36 GW**	6 (5; 7)	6 (5; 8)	0.002
**NUTRITION SCORE**			
**12 GW**	0 (−2; 3)	0 (−2; 3)	0.709
**24 GW**	1 (−1; 4)	3 (1; 5)	0.000
**36 GW**	4 (1; 7)	5 (2: 7)	0.002
**PHYSICAL ACTIVITY SCORE**			
**12 GW**	−2 (−3; −1)	−2 (−3; −1)	0.373
**24 GW**	−2 (−2; −1)	−2 (−2; −1)	0.798
**36 GW**	−2 (−2; −1)	−2 (−2; −1)	0.015

Data are Median (IQR) or number (%). blood pressure (BP); newborn (NB); Mediterranean Diet Adherence Screener (MEDAS). Physical Activity Score, (Walking daily (>5 days ⁄ week) Score 0: At least 30 min. Score +1, if >60 min. Score −1, if <30 min. Climbing stairs (floors ⁄ day, >5 days a week): Score 0, between 4 and 16; Score +1, >16; Score −1: <4).
